# Knowledge-based analysis of genetic associations of rheumatoid arthritis to inform studies searching for pleiotropic genes: a literature review and network analysis

**DOI:** 10.1186/s13075-015-0715-1

**Published:** 2015-08-08

**Authors:** Weiying Zheng, Shaoqi Rao

**Affiliations:** College of Biomedical Engineering, Capital Medical University, 10 Xitoutiao Youanmen Fengtai, Beijing, 100069 People’s Republic of China; Institute of Medical Systems Biology and School of Public Health, Guangdong Medical College, 1 Xin Cheng Avenue, Songshan Lake, Dongguan, 523808 Guangdong People’s Republic of China

## Abstract

**Introduction:**

Pleiotropy describes the genetic effect of a single gene on multiple phenotypic traits. Gene variants directly affect the normal processes of a series of physiological and biochemical reactions, and therefore cause a variety of diseases traits to be changed accordingly. Moreover, a shared genetic susceptibility mechanism may exist between different diseases. Therefore, shared genes, with pleiotropic effects, are important to understand the sharing pathogenesis and hence the mechanisms underlying comorbidity.

**Methods:**

In this study, we proposed combining genome-wide association studies (GWAS) and public knowledge databases to search for potential pleiotropic genes associated with rheumatoid arthritis (RA) and eight other related diseases. Here, a GWAS-based network analysis is used to recognize risk genes significantly associated with RA. These RA risk genes are re-extracted as potential pleiotropic genes if they have been proved to be susceptible genes for at least one of eight other diseases in the OMIM or PubMed databases.

**Results:**

In total, we extracted 116 potential functional pleiotropic genes for RA and eight other diseases, including five hub pleiotropic genes, *BTNL2*, *HLA-DRA*, *NOTCH4*, *TNXB*, and *C6orf10*, where *BTNL2*, *NOTCH4*, and *C6orf10* are novel pleiotropic genes identified by our analysis.

**Conclusions:**

This study demonstrates that pleiotropy is a common property of genes associated with disease traits. Our results ascertained the shared genetic risk profiles that predisposed individuals to RA and other diseases, which could have implications for identification of molecular targets for drug development, and classification of diseases.

**Electronic supplementary material:**

The online version of this article (doi:10.1186/s13075-015-0715-1) contains supplementary material, which is available to authorized users.

## Introduction

Rheumatoid arthritis (RA) is one of the most common systemic autoimmune diseases, characterized by synovial inflammation and hyperplasia, autoantibody production, cartilage and bone destruction, and systemic features, including cardiovascular, pulmonary, psychological, and skeletal disorders [1]. Parikh-Patel et al. [2] reported that among RA patients in California, males had significantly higher risks of lung neoplasm, but a lower risk of prostate neoplasm; while females had a significantly reduced risk for breast neoplasm. In addition, these diseases were race-specific and Hispanics had increased risks of leukemia and lung neoplasms. It is also recognized that some patients with systemic lupus erythematosus (SLE) will develop a symmetrical polyarthritis while others might not experience any arthritis at all [3]. Lewder [4] reckoned that 15–20 % of patients with psoriasis would develop arthropathies, which shared some common features with reactive arthritis. Several recent studies indicated that autoimmune disorders increase the likelihood for prostate neoplasm [5] and lung neoplasm [6, 7]. Khurana et al. [8] showed a significant positive association between RA and the development of lung neoplasm in the veteran population. Isomäki et al. [9] reported that the incidence of leukemia was significantly higher in patients with rheumatoid arthritis than in the general male population.

The chronic, debilitating, autoimmune nature of RA affects the patient directly or indirectly in almost all organ systems, from cardiovascular problems and infections to depression and gastrointestinal ulcers. On average, the established RA patient has two or more comorbid conditions [10]. Causes of death in RA patients are cardiovascular disease in 31 %, respiratory disease in 22 %, solid tumors in 20 %, cerebrovascular disease in 10 % and other reasons in 17 % [11, 12]. Thus, studies to examine the molecular bridge linking RA and numerous related disease (for examples, breast neoplasm, Alzheimer disease, diabetes mellitus, type 1 (T1D), prostate neoplasm, lung neoplasm, psoriasis, SLE and leukemia, investigated by the present study) are in high demand.

Genomic variation can influence disease susceptibility, disease progression, the risk of specific outcomes, or the individual’s response to therapy. Most molecular genetic studies focus on the identification of genes involved in a single human disease. To date, approximately 1,800 genes have been identified that are mutated in human diseases [13]. However, there are limited attempts to study multiple diseases together to discover the molecular bridges between them. In fact, the molecular mechanisms for complex human diseases could be more sophisticated than what we are imaging. On the one hand, a single disease phenotype can be the result of mutations in many different genes, the so-called genetic heterogeneity, and on the other hand one gene also can affect several different phenotypes, namely genetic pleiotropy. In definition, pleiotropy describes the genetic effect of a single gene on multiple phenotypic traits, which occurs when a new mutation in the gene may have an effect on some or all traits simultaneously or may cause different pathological effects in complex human diseases [14–16].

Pleiotropy in diseases often occurs when an impaired gene generates pathological effects on some molecular-associated diseases simultaneously. For example, the gene *PTPN22* showed pleiotropic effects in multiple autoimmune diseases including T1D, SLE and RA [17]. Furthermore, one clear finding to emerge from the published genetic studies of autoimmunity was that different autoimmune diseases shared common susceptibility loci. The *HLA* region was well known for being associated with several autoimmune diseases including T1D, multiple sclerosis (MS), RA as well as others [18–20].

Recently, Chavali et al. [16] described a method for estimating the pleiotropic effects of human disease genes from network properties. Sivakumaran et al. [21] presented a systematic review of pleiotropy among single nucleotide polymorphisms (SNPs) and genes and found that pleiotropic links between common complex diseases and traits occurred more often than expected. Goh et al. [13] performed a comprehensive analysis of Online Mendelian Inheritance in Man (OMIM) for human traits and found several hundred genes of pleiotropic nature. And this gene list was expected to grow fast with increasing applications of the large-scale genome-wide association studies (GWAS) approach [22].

The GWAS approach analyzes SNPs across the whole genome to robustly identify key inherited genetic variations that have critical but as yet largely uncharacteristic roles in development of human diseases. The success of GWAS has opened a wide new horizon for exploration and highlighted the complicated genomic architecture of disease susceptibility. However, there are few attempts to use the GWAS approach to explore the shared (pleiotropic) genetic factors, which may give clues to underlying etiological links between these diseases and pinpoint potential directions and practical recommendations for future research in this field.

In the present study, we provided an integrated approach to combine GWAS and public knowledge databases, seeking for pleiotropic genes that link RA to eight other complex diseases. Here, the GWAS approach was used to obtain single risk SNPs from the Wellcome Trust Case Control Consortium (WTCCC) dataset, and the acquired SNPs were mapped to genes to extract risk genes to RA. Such genes were defined as pleiotropic genes if they had been proved to be also the susceptible genes for at least one of eight other diseases by using public knowledge databases PubMed or OMIM. To extract the disease-related pleiotropic functional modules and hub genes, we further used an interactive tree-model and logistic regression model to construct a SNP-SNP network for RA. In total, we extracted 116 pleiotropic genes, including five hub pleiotropic genes: *BTNL2*, *HLA-DRA*, *NOTCH4*, *TNXB* and *C6orf10*, where *BTNL2*, *NOTCH4*, and *C6orf10* were novel ones identified by this study. This exploratory study provided promising clues to inform experimental studies searching for pleiotropic genes.

## Materials and methods

### Data source

Genome-wide SNP data were provided by WTCCC, which contains SNP genotypic data for seven common diseases and the shared control. The present study focused on the analysis of the GWAS data for RA, which included 1,860 cases and 2,938 controls. Prior to association analysis, some SNPs were excluded due to significant deviations from the Hardy-Weinberg equilibrium (*P* <5 × 10^−5^), or having a high rate of missing data (genotyping efficiency <95 %), or minor allele frequency (MAF) <1 %.

### Extracting risk SNPs associated with RA

First, genome-wide single-point SNP association analysis was performed by two methods, the interactive tree-model analysis and conventional logistic regression. Multiple testing corrections were performed, and the *P* values were adjusted by using the false discovery rate (FDR) method, with the family-wise error rate of *P* <0.05. This analysis was implemented by using HelixTree software (see [23]).

### Identifying pleiotropic genes for RA and other diseases

To mine high-risk genes linked with RA, SNPs were mapped to genes using the SNPnexus tool (see [24]). To extract pleiotropic actions of the identified genes for RA, we manually selected those genes that had been proved to be associated with at least one of eight other diseases by literature reviews of the PubMed or OMIM database.

### Identifying hub SNPs

To understand the functional mechanisms involved in RA pathogenesis, we further analyzed genetic factors along with biological network information [25]. Significant SNP-SNP interactions (nominal *P* <0.01) were detected by using the interactive tree-based method. Then, a logistic regression model was used to estimate their odds ratios and 95 % CIs (confidence intervals). All the significant SNP-SNP interaction pairs contributing to RA were used to construct a disease-specific SNP-SNP network for RA. In the network, the nodes represented SNPs, and the links between SNPs suggest their synergistic actions contributing to RA. As a network measure, connectivity (the number of links) was used to measure importance of a hub node. To obtain significant hub nodes, we assumed that in a random network, connectivity followed a Poisson distribution in a random network [26, 27]. We used the following formula to assess whether a node could be categorized as a hub node. Suppose that *p* was the probability of connecting any two nodes in a random network with *n* nodes, the probability of connectivity of equal or larger than *t* was as follows:

$$ p\left(x\ge t\right)=1-p\left(x<t\right)=1-{\displaystyle \sum_{k=0}^{t-1}\frac{\lambda^k{e}^{-\lambda }}{k!}},\;\left(\lambda =n{P}_1,{P}_1=m/{C}_n^2\right), $$

Where *m* is the number of interacting SNP pairs contained in the disease-specific SNP network. We considered a SNP with >5 connections (*P* = 0.01) in a random network as a rare event under the null hypothesis that *n* nodes (SNPs) were connected randomly. The probability of this rare event was taken as a threshold, and a node was considered a hub SNP when its *P* value was smaller than the threshold.

### Mining hub pleiotropic genes

To mine the disease-related pleiotropic functional modules, the SNP network was first turned into a gene network by mapping SNPs to genes using the dbSNP database. We used the following rule for mapping SNPs onto genes, i.e., to see whether a SNP is located within a gene or the untranslated regions (UTRs) of this gene. SNPs that were mapped onto multiple genes were assigned to a single gene according to the following hierarchy: coding > intronic > 5′ UTR > 3′ UTR > 5′ upstream > 3′ upstream. This strategy can avoid issues with a weighted inflation induced by genes having different numbers of SNPs [28]. All the significant gene-gene interaction was utilized to construct a gene network. In both SNP-SNP and gene-gene interaction assays, we chose to use a nominal level of *P* <0.01 as the cutoff to identify important SNP pairs or gene pairs because no suitable methodologies for adjusting for multiple correlated tests of massive SNP pairs (or gene pairs) were available, which may have led to false positives. However, in the following step for identifying hub pleiotropic genes, we may alleviate this issue by using evidence from the network analysis and literature reviews.

## Results

### Identifying pleiotropic genes for RA and related diseases

In single-point genome-wide association analysis of 459,236 autosomal SNP markers, 2046 SNPs were found significantly associated with RA. These SNPs were mapped to 502 RA risk genes (See the Table S1 in Additional file 1 and Table S2 in Additional file 2).

To confirm their pleiotropic effects, we tried to find evidence from OMIM or PubMed by literature reviews to support that these genes were associated with at least one of eight other diseases (i.e., breast neoplasm, Alzheimer disease, T1D, prostate neoplasm, lung neoplasm, psoriasis, SLE and leukemia). We extracted a total of 116 pleiotropic genes (corresponding to 205 SNPs), see Additional file 3 for details. Their pleiotropic associations with multiple diseases were summarized in Additional file 4.

In these 116 genes, *HLA-DRB1* was discovered first and is still by far the strongest genetic link to RA. It was estimated that this *HLA* locus contributed about 30 % of the overall familial RA risk [29]; *PTPN22*, a lymphocyte-specific nonreceptor tyrosine phosphatase involved in regulation of activation threshold of lymphocytes, was the second most contributed genetic link to RA. *PTPN22* also represented a strong susceptibility gene, which was shared by many autoimmune diseases such as T1D, psoriasis, and SLE. This suggested the presence of common genetic factors that predisposed to multiple autoimmunity diseases. Besides, pleiotropic genes *HLA-DRB1, PTPN22, AFF3* and *IL2RA* were all included in a T cell activation pathway. In this pathway, some predisposing T cell repertoire selection, antigen presentation, or alteration in peptide affinity had a role in promoting autoreactive adaptive immune responses [1]. There were studies indicating that intracellular machinery was affected in T cells of RA patients, which might alter the behavior of T cells during activation. Different therapeutic approaches may modulate the abnormal T cell functions [30]. Protein tyrosine phosphatases (*PTPN22*) were the critical regulators of T cell signal transduction. In conjunction with protein tyrosine kinases, protein tyrosine phosphatases regulated the reversible phosphorylation of tyrosine residues and thereby played important roles in many diverse aspects of T cell physiology. Abnormalities in tyrosine phosphorylation had turned out to be involved in the pathogenesis of numerous human diseases, from autoimmunity to cancer [31].

More recently, we occasionally found that one of the 116 genes, *TNFAIP3*, should be a susceptibility factor for RA in the northern Chinese Han population [32] and its pleiotropic association with SLE and RA in the Korean population were also reported [33]. *TNFAIP3* played a major role in downregulating TNF-induced nuclear factor kappa B (NF-κB) activation. As is well known, acute inflammation is a part of the defense response; chronic inflammation can lead to cancer, diabetes, arthritis, Alzheimer’s disease, pulmonary, and neurological diseases. Regulation of NF-κB helped in explaining the linkages of inflammation and cancer at the molecular level. Transcription factor NF-κB mainly regulated the expression of several proinflammatory gene products TNF and its superfamily members [34]. NF-κB was constitutively active in most tumors and was induced by carcinogens, tumor promoters, carcinogenic viral proteins, chemotherapeutic agents, and γ-irradiation. Hence, anti-inflammatory agents, which suppressed NF-κB or NF-κB-regulated products, should have a potential in both the prevention and treatment of cancer [35].

### Identifying pleiotropic SNP-SNP interactions and hub genes for RA and related diseases

**Fig. 1:**
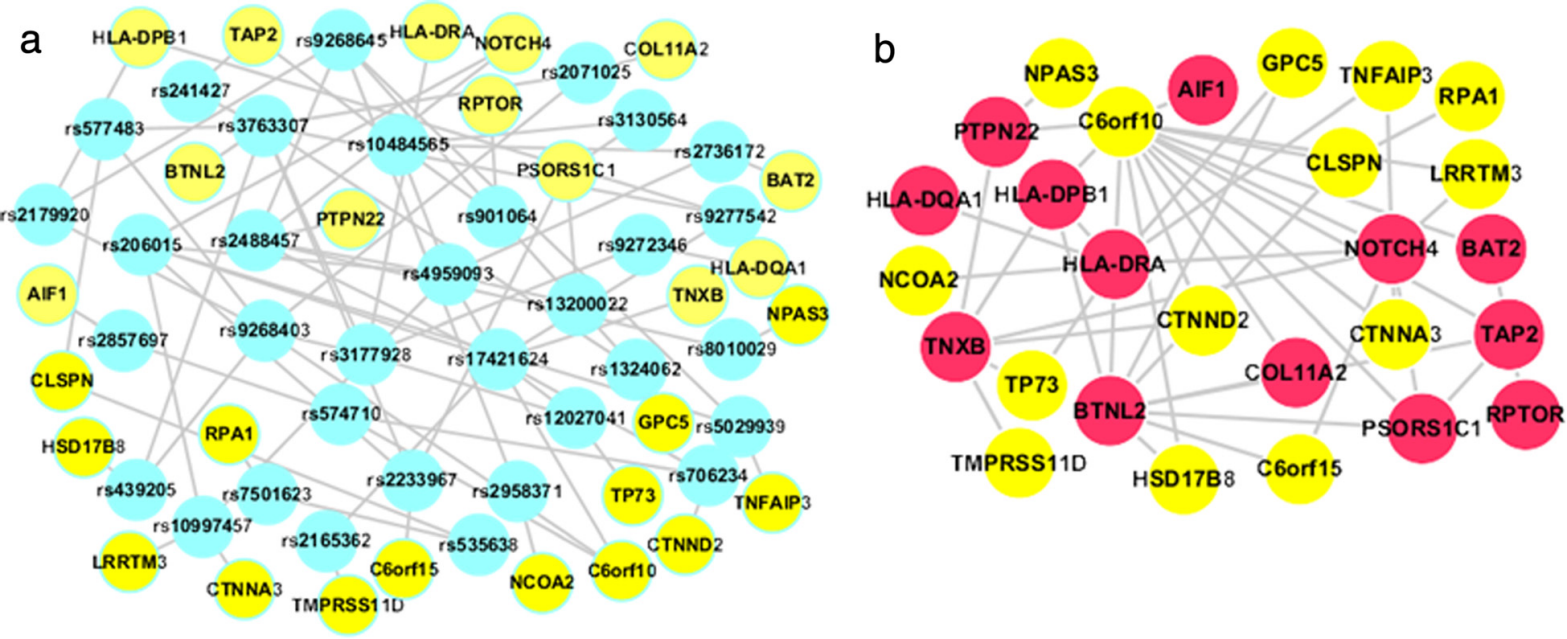
A pleiotropic interaction network of RA. **a** The pleiotropic SNP-SNP interaction network; **b** the derived gene-gene interaction network. In **b**, 13 *red nodes* are RA-susceptible genes, and 14 *yellow nodes* are susceptible genes for one or more of eight other diseases. *SNP* single nucleotide polymorphism, *RA* rheumatoid arthritis

These 205 pleiotropic SNPs were subject to an epistasis analysis using the above mentioned two approaches. First, 127 pairs of SNP interactions (*P* <0.01) were detected by using classification tree-based analysis. Then, logistic regression models were used to compute the odds ratios and their 95 % CIs for these SNP-SNP interactions, confirming that 46 SNP pairs were significant interactions (*P* <0.01) (see Additional file 5). A SNP-SNP interaction network was constructed as shown in Fig. 1. By turning into a gene-gene network, 27 pleiotropic genes were included in the network and five hub genes were extracted by using a Poisson-based test of connectivity: *C6orf10* (degree = 14, P = 1.1 × 10^−11^), *BTNL2* and *HLA-DRA* (degree = 9, *P* = 1.3 × 10^−5^), *NOTCH4* (degree = 8, *P* = 2.3 × 10^−5^), *TNXB* (degree = 6, *P* = 8.6 × 10^−3^).

*HLA-DRA*, as one of the genes contained in major histocompatibility complex (MHC) region, had been discussed in the previous section. Involvement of the MHC region in chromosome 6p21 was in no doubt for most autoimmune diseases [36].

*NOTCH4* encoded a receptor protein that was involved in multiple cellular processes, such as cell differentiation, proliferation and apoptosis. OMIM recorded that *NOTCH4* was highly related to schizophrenia. However, we also found its association with RA, breast neoplasm, and Alzheimer disease in PubMed. AlFadhli [37] was the first one reporting a statistically significant association between *NOTCH4* and RA. Furthermore, SNP hla58, located in the intron of *NOTCH4* gene was observed to be in strong linkage disequilibrium with *HLA-DRB1* allele in RA [38]. Also, *NOTCH4* was a potential new therapeutic target for triple-negative breast neoplasm [39]. An mRNA transcript encoding the intracellular domain of *NOTCH4* was detected in two human breast neoplasm lines [40]. Furthermore, *NOTCH4* may play important roles in the pathogenesis of Alzheimer disease in the Japanese population and in the United Kingdom population [41, 42].

Published reports for *TNXB* gene were relatively limited. *TNXB* gene, located near the MHC class III region (6p21.3), had anti-adhesive effects, and functions in matrix maturation in connective tissues [43]. *TNXB* gene encoded an extracellular matrix protein, tenascin XB, which regulated collagen synthesis and deposition [44, 45]. In OMIM, *TNXB* was a pleiotropic gene of Ehlers-Danlos syndrome and Vesicoureteral reflux diseases. Furthermore, Rupert et al. [46] reported an unequal crossover between *RCCX* modules of human *MHC* leading to the presence of a *CYP21B* gene and a tenascin *TNXB/TNXA-RP2* recombinant in patients with juvenile RA. In other research, *BTNL2, C6orf10, NOTCH4, TAP2*, and *TNXB* were all identified as the novel RA-associated genes [47]. Not only that, a study by Kamatani and colleagues [45] identified rs3130342 in the 50 flanking region of the *TNXB* as a possible candidate gene susceptible to SLE in the Japanese population.

*BTNL2* gene in OMIM was labeled as a sarcoidosis disease susceptibility gene. However, *BTNL2* (rs3817963) was reported as a new susceptibility locus of lung adenocarcinoma as well, the most common histologic type of lung neoplasm in the Japanese population [48]. As reported, patients with RA more often had lymphomas and lung tumors with the standardized incidence ratios of 2.1 and 1.6 respectively [49]. Functional variant rs2076530 of the *BTNL2* gene was identified conferring susceptibility to the autoimmune diseases T1D, RA, and SLE (G allele was linked to T1D and RA, and the A allele associated with SLE) [50]. Furthermore, there were new results implicating *BTNL2* as a novel prostate neoplasm-related gene [51].

**Fig. 2:**
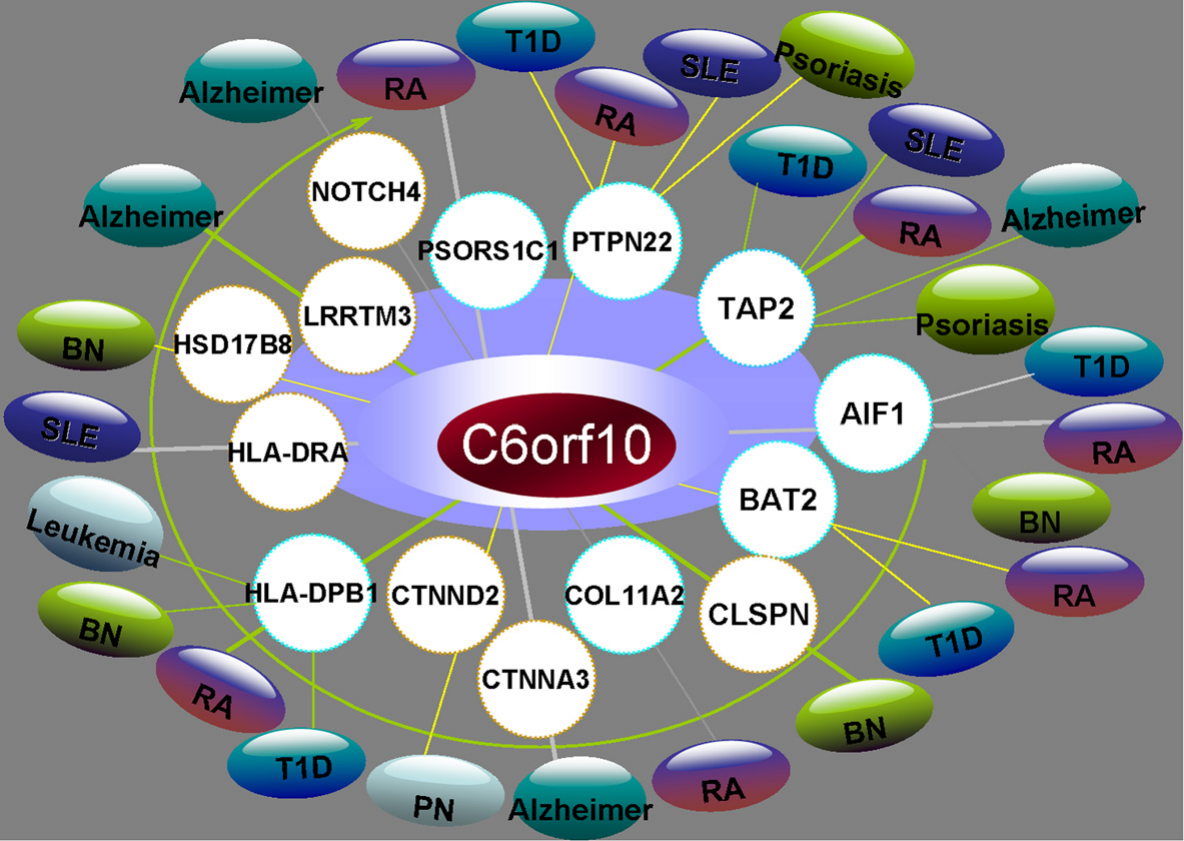
The gene-disease network of *C6orf10* and its interaction genes

*C6orf10* gene had not yet been included in OMIM. *C6orf10* lies between *NOTCH4* and *BTNL2*. However, *C6orf10* was a completely new pleiotropic gene that had not been reported before. In our analysis, 12 SNPs located within *C6orf10* (rs9268402, rs9268403, rs574710, rs4959093, rs3129932, rs3132959, rs9368716, rs2894249, rs3129934, rs3129933, rs910050, rs9268208) were significantly related to RA. *C6orf10* was one of five hub disease genes in our constructed pleiotropic genetic network. There were 14 genes interacting with *C6orf10*, indicating its strong risk for multiple diseases. We provided the associated diseases with 14 genes (see Additional file 6) and exhibited a corresponding gene-disease network to predict the pleiotropic function of *C6orf10* (see Fig. 2).

We manually searched for literature in PubMed for *C6orf10* to explore its function. Table 1 summarizes the related diseases of *C6orf10* reported to date.

**Table 1:** The list of gene *C6orf10* related diseases, as a novel RA pleiotropic gene identified by GWAS analysis

SNPs	Population	Disease relevance	PubMed ID
rs1265777, rs574710, rs539703, rs2894249	British	RA	20018019
rs6910071, rs9391858, rs10484560, rs6910071	North American	RA	20018025, 20018077, 20018006, 21592391
rs2395148	Canadian, U.S.	PBC	19458352
rs2073048, rs28732201	European, Chinese Han	Psoriasis	19680446, 20692714
rs926070, rs2073044	Cleveland	Leukemia	20460636
rs3130320		SLE	21408207
rs3117103, rs7746019	European	SLE	19851445
rs7775397		NL	22031281
rs7758128	European	Vitiligo	21326295
rs485774	U.S., Canada	LOAD	22245343
rs498422	Chinese Han	NOA	22541561
rs2050190	Utah	CAD	22703881
DG6S398, D6S2889	Caucasians	T1D	19143811
rs9391858	British	T1D	20549515
rs3129934	European	MS	19010793

*GWAS* genome-wide association study, *RA* rheumatoid arthritis, *PBC* primary biliary cirrhosis, *Leukemia* T cell large granular lymphocyte leukemia, *SLE* systemic lupus erythematosus, *NL* neonatal lupus, *Vitiligo* generalized vitiligo, *LOAD* late-onset Alzheimer disease, *NOA* nonobstructive azoospermia, *CAD* coronary artery disease, *T1D* diabetes mellitus, type 1, *MS* multiple sclerosis

Formerly, *C6orf10* was only known as testis-specific basic protein, and for which no transcripts had been assigned to date. It had been known that expression of this gene in keratinocytes was induced by exposure to tumor necrosis factor alpha (TNF-α)*.* TNF-α*-*directed therapy had turned out to be valuable in the treatment of patients with refractory RA [52]. TNF-α inhibitors had been a cornerstone in the treatment of several chronic inflammatory diseases. Among patients with RA and a history of breast neoplasm, those who started a TNF-α-inhibitor treatment did not experience more breast neoplasm recurrences than patients with RA treated otherwise. TNF-α inhibitors might impact the risk of cancer development, or modify the risk of recurrence of previous cancers [53].

As non-HLA genes, we hypothesized that *NOTCH4*, *TNXB*, *BTNL2,* and *C6orf10* all would be good candidates for further clinical and laboratory studies. We postulated that to achieve success in the study of RA (as well as other diseases), it was necessary to take into account the multivariate nature of these diseases.

## Discussion

GWAS analysis, by searching the entire human genome for association, is a promising approach to unravel the genetic basis of complex genetic diseases. Analysis of omic SNPs permitted determining relationships between genotypes and phenotypes, identification of risk SNPs and genes related to disease [54]. In this study, we presented an integrative approach to combine network analysis of GWAS data with literature reviews in order to recognize pleiotropic genes linked to RA and related diseases. In total, we found 116 genes harboring variants associated with RA and eight other diseases. In order to verify these findings, we sought supportive evidence by reviewing two additional databases for disease genes (MalaCards and HuGENavigator).

MalaCards [55] is an integrated web database of human maladies and their annotations, which included 64 data sources. In MalaCards, using GeneCards Suite and keywords of eight other diseases (breast neoplasm, Alzheimer disease, T1D, prostate neoplasm, lung neoplasm, psoriasis, SLE and leukemia), we compared 116 pleiotropic genes identified in this study with the disease genes related to the diseases. In total, 87 of the 116 pleiotropic genes were found to be related to the eight diseases. However, we found that some disease-related genes confirmed in the OMIM database, such as T1D-associated genes (*FOXP3, HNF1A, OAS1, ITPR3, PTPN22*) and SLE-associated genes (*BANK1*) were not included in MalaCards database. Thus, this verification using only MalaCards was incomplete and inadequate. So, we chose another database, HuGENavigator, for further comparison.

**Fig. 3:**
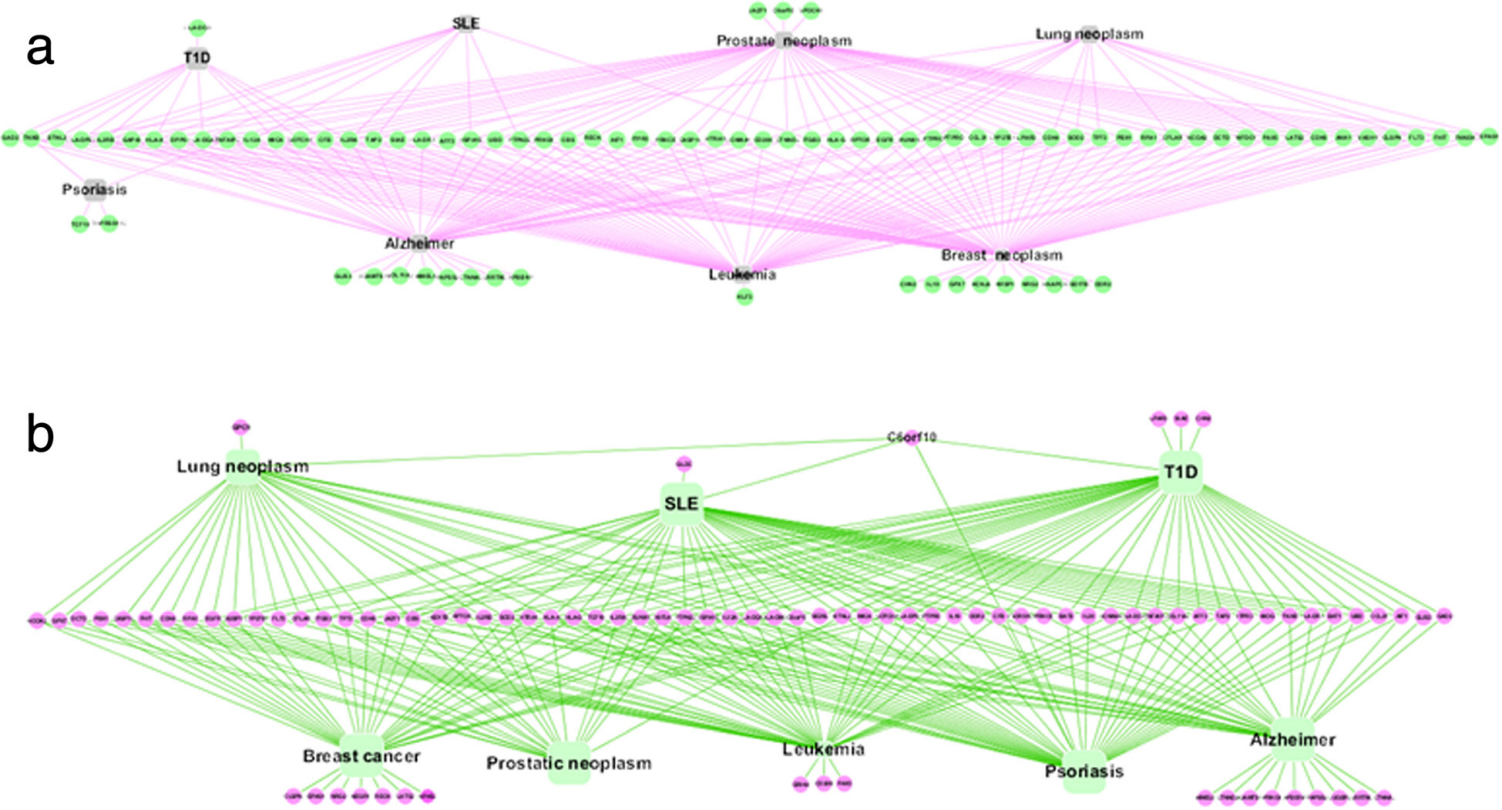
RA pleiotropic genes replicated in MalaCards and HuGENavigator databases. **a** Pleiotropic genes of RA in MalaCards. **b** Pleiotropic genes of RA in HuGENavigator. *RA* rheumatoid arthritis

HuGENavigator [56] is an integrated, searchable knowledge base of genetic associations and human genome epidemiology. In HuGENavigator, we performed a similar analysis, and found that a total of 89 pleiotropic genes were replicated by HuGENavigator (i.e., also related with the eight diseases). The list of genes replicated in MalaCards and HuGENavigator is provided in Additional file 7. Moreover, we found that *C6orf10* was also proved a pleiotropic risk gene for SLE, T1D, psoriasis and lung neoplasm in HuGENavigator. For comprehensive results of this additional analysis, see Fig. 3.

Among these 116 pleiotropic genes, most of our extracted pleiotropic genes were found to link with multiple diseases, while others are novel, requiring further studies (either experimental or computational) to verify them prior to applying these findings to more practical settings.

We believed that examples of pleiotropy would accumulate over time, for it was already clear that pleiotropy was a common property of genes and SNPs associated with disease traits [21]. The clustering of autoimmune disorders within families and/or individuals also provided supportive evidence for these shared genetic factors [29].

Pleiotropic genes built up a bridge between RA and eight other diseases; they also would shed light on some studies of the inflammation-cancer link. Clinical studies suggested that persistent inflammation functions as a driving force in the journey to cancer. The possible mechanisms by which inflammation could contribute to carcinogenesis include induction of genomic instability, alterations in epigenetic events and subsequent inappropriate gene expression, enhanced proliferation of initiated cells and resistance to apoptosis [57]. Pleiotropic genes and their products should play an important role in this process. Also there was a study that clearly pointed to the importance of anti-inflammatory drugs in preventing the initiation and progression of both gastrointestinal and other solid organ cancers (including lung and prostate), and suggested that inflammation might be an underlying cause of cancer even in tumor types that had not been traditionally considered to originate within chronically inflamed tissues [58].

Despite that this study provided a pioneering approach to analyze large-scale SNP omic data and to fusing data from rich knowledge databases, we should recognize the limitations of this study in exploring pleiotropic mechanisms between complex diseases. First, because a single GWAS dataset was used for analyzing RA, we believe that only a small proportion of pleiotropic genes were identified, and perhaps a long list of genes with modest effects remains to be found in further independent studies. The omic SNP data, provided by WTCCC, are for Caucasians, whether these findings could be extended to other nationalities remains unclear. Furthermore, due to lack of information, this study did not model several important epidemiological covariates like gender, age and so on, which may have an impact on this pleiotropic analysis. Second, currently accumulated knowledge about genes involved in complex diseases, especially of their pleiotropic effects, was incomplete and fragmented, and part of this analysis that relied on knowledge mining suffers from this limitation. Finally, although we tried our best to control Type I errors in various steps toward identification of pleiotropic genes, whether the overall Type I error was well controlled remains unclear. In this sense, we view our analysis exploratory in nature.

In spite of the encouraging successes in finding pleiotropic genes for RA achieved by this study, further deeper studies of their detailed molecular etiology or links for these correlated diseases are certainly required. We deeply believe that the pleiotropic genes identified by this study only represent a tip of the iceberg in the genetic architectures for complex diseases. Further studies, with more sophisticated designs and more involved multivariate analysis, are called for to directly test the hypothesis that a pleiotropic gene has a duplicated genetic effect on multiple disease phenotypes, followed by experimental or clinical validation.

## Conclusions

Identification of pleiotropic genes is fundamental to reveal the underlying genetic links that connect multiple related diseases at the molecular level. In this study, we provided an integrative approach to combine network analysis of GWAS data with literature reviews in order to identify pleiotropic genes for complex diseases. Application to a GWAS dataset for RA identified a list of potential pleiotropic genes, which may be valuable clues for experimental studies to decipher the molecular mechanisms underlying these pleiotropisms.

## Additional files

Additional file 1:
**The list of susceptible SNPs involved in RA by GWAS.** (XLS 188 kb) Additional file 2:
**The list of susceptible genes involved in RA by GWAS.** (XLS 251 kb) Additional file 3:
**The list of significant pleiotropic SNPs and genes that are involved in RA and one or more other diseases.** (XLS 61 kb) Additional file 4:
**Risk RA genes were proved to be susceptible to RA and eight other diseases respectively confirmed by the PubMed literature database.** (XLS 35 kb) Additional file 5:
**The list of pleiotropic SNP-SNP interactions that are involved in RA and one or more other diseases.** (XLS 23 kb) Additional file 6:
**The interaction genes with** ***C6orf10*** **in gene networks and their associated diseases.** (XLS 21 kb) Additional file 7:
**The list of RA pleiotropic genes replicated in MalaCards and HuGENavigator.** (XLS 38 kb)

## Abbreviations

BANK1: B-cell scaffold protein with ankyrin repeats 1
BTNL2: butyrophilin-like 2
C6orf10: chromosome 6 open reading frame 10
FOXP3: forkhead box P3
GWAS: genome-wide association study
HLA: human leukocyte antigen
HLA-DRA: major histocompatibility complex, class II, DR alpha
HNF1A: HNF1 homeobox A
ITPR3: inositol 1,4,5-trisphosphate receptor, type 3
MHC: major histocompatibility complex
MS: multiple sclerosis
NF-κB: nuclear factor kappa B
NOTCH4: neurogenic locus notch homolog 4
OAS1: 2′-5′-oligoadenylate synthetase 1, 40/46 kDa
OMIM: Online Mendelian Inheritance in Man
PTPN22: tyrosine phosphatase nonreceptor 22
RA: rheumatoid arthritis
SLE: systemic lupus erythematosus
SNP: single nucleotide polymorphism
T1D: diabetes mellitus, type 1
TNFAIP3: tumor necrosis factor alpha-induced protein 3
TNF-α: tumor necrosis factor alpha
TNXB: tenascin XB
UTR: untranslated region
WTCCC: Wellcome Trust Case Control Consortium
